# Integrated Bio-Entity Network: A System for Biological Knowledge Discovery

**DOI:** 10.1371/journal.pone.0021474

**Published:** 2011-06-27

**Authors:** Lindsey Bell, Rajesh Chowdhary, Jun S. Liu, Xufeng Niu, Jinfeng Zhang

**Affiliations:** 1 Department of Statistics, Florida State University, Tallahassee, Florida, United States of America; 2 Marshfield Clinic-Marshfield Center, MCRF-BIRC, Marshfield, Wisconsin, United States of America; 3 Department of Statistics, Harvard University, Cambridge, Massachusetts, United States of America; University of Georgia, United States of America

## Abstract

A significant part of our biological knowledge is centered on relationships between biological entities (bio-entities) such as proteins, genes, small molecules, pathways, gene ontology (GO) terms and diseases. Accumulated at an increasing speed, the information on bio-entity relationships is archived in different forms at scattered places. Most of such information is buried in scientific literature as unstructured text. Organizing heterogeneous information in a structured form not only facilitates study of biological systems using integrative approaches, but also allows discovery of new knowledge in an automatic and systematic way. In this study, we performed a large scale integration of bio-entity relationship information from both databases containing manually annotated, structured information and automatic information extraction of unstructured text in scientific literature. The relationship information we integrated in this study includes protein–protein interactions, protein/gene regulations, protein–small molecule interactions, protein–GO relationships, protein–pathway relationships, and pathway–disease relationships. The relationship information is organized in a graph data structure, named integrated bio-entity network (IBN), where the vertices are the bio-entities and edges represent their relationships. Under this framework, graph theoretic algorithms can be designed to perform various knowledge discovery tasks. We designed breadth-first search with pruning (BFSP) and most probable path (MPP) algorithms to automatically generate hypotheses—the indirect relationships with high probabilities in the network. We show that IBN can be used to generate plausible hypotheses, which not only help to better understand the complex interactions in biological systems, but also provide guidance for experimental designs.

## Introduction

Relationships among bio-entities, such as proteins, genes, diseases, biological pathways and gene ontology (GO) terms, constitute a significant part of our biological knowledge. Protein-protein interactions, for example, play central roles in almost all biological processes and are indispensable for our understanding of the mechanisms of biological processes and for development of drugs[1]. Manual annotation has been used to extract such information from scientific literature and deposit it into databases as structured form[2], [3], [4], [5], [6], [7], [8], [9], [10], [11], [12], [13], [14], [15], [16], [17], [18], [19]. However, manual annotation is quite time and resource consuming and it has become more and more difficult to keep pace with the ever increasing publications in biomedical science. In recent years, computational methods have been developed to automatically extract molecular interaction information and other bio-entity relationships from literature and been used to assist the human annotators in building databases[20], [21], [22], [23], [24], [25], [26], [27], [28], [29], [30], [31], [32], [33], [34], [35], [36], [37], [38], [39], [40], [41]. When information is located at different places, it is not convenient to conduct research that requires integration of separated pieces of information. Ideally, one would like to have heterogeneous information integrated into structured forms that allow both convenient retrieval and more complicated computations on such information. Studies have been initialized toward such goals for some important types of biological information. For instance, the National Center for Biological Information (NCBI) has built databases such as Entrez Gene[42], which stores information from both curation and automated integration of data from NCBI's Reference Sequence project (RefSeq)[43] and other databases. Gene Ontology database[44], which documents biological terms such as molecular functions, biological processes, and cellular locations, has also been linked with proteins that are related to the corresponding terms[26], [45]. Integration of information is critical to understanding biology at system level and accelerating scientific discoveries to keep up with the rapidly increasing rate of new biological information being produced.

Integration of information from different sources/domains makes it possible to discovery new knowledge through automatic hypothesis generation. Knowledge discovery has been an active topic[22], [23] since Swanson's pioneer work more than 30 years ago[46]. The concept is rather simple: If there are relationships between A and B, and B and C, then one can hypothesize a possible relationship between A and C. However, when the two known relationships are published at different places, it can be difficult to identify them and make the connection. Some literature-based discovery (LBD) systems have been developed in the past based on this idea such as BITOLA[47], iridescent[48], Manjal[49], LitLinker[50] and CoPub Discovery[51]. They aim to find the hidden relationships automatically through information extraction and integration. There are several drawbacks for the previous systems to be used in inferring biological relationships, especially molecular interaction information, which plays a central role in these relationships. Firstly, they did not integrate manually annotated and structured (MAS) information in publically accessible databases such as protein interaction databases[5], [11], [13]. A knowledge discovery system should distinguish what has been known from what may be new. In fact, some of the information discovered by these early systems may have already been deposited as MAS information in databases. In addition, these systems likely miss some MAS information for a given query, which would have been easily found by searching a database. Integrating MAS information can also greatly help knowledge discovery. For example, when inferring relationship between A and C (relationship R_1_) using two relationships A and B (R_2_), and B and C (R_3_), the probability of R_1_ can be more accurately inferred if R_2_ and R_3_ are true. Secondly, those previous knowledge discovery methods use co-occurrence of terms in abstracts to infer their relationships. Such approach is not effective for inferring molecular interaction information since it has been shown that even using co-occurrence in the same sentence, the false positive rate can be very high[38]. This is because many molecule names co-occur in the same sentence but do not interact with each other. Thirdly, the earlier systems do not explicitly consider the type of relationship between two bio-entities. For instance, words like *inhibit*, *activate*, and *phosphorylate* express different types of interaction information. Without incorporating the information on the type of interactions, a knowledge discovery system tends to return a large number of false positives (see an example in Result, case study 3).

Several difficulties need to be overcome to make automatic knowledge discovery systems effective tools in biomedical research. Firstly, information from different sources/domains needs to be integrated in a structured way. This is highly nontrivial due to the difference in data organizations and discrepancy in information from different databases caused by inevitable annotation errors or inherent ambiguity/uncertainty of certain information. Secondly, relationship information needs to be well annotated to allow for effective *information flow* from one bio-entity to another. For example, in protein-protein interaction databases, how proteins interact with other proteins, such as *inhibit*, *regulate*, *phosphorylate* etc, is usually not well documented. Other information such as the directionality of the interaction[36], the cellular location of the interactions and the function of the interactions are seldom provided despite that such information can be very important for the understanding and use of the interaction information in research. Thirdly, with large volumes of information, false positives will be a major issue for automatically generated hypotheses. Ranking the hypotheses or providing confidence levels would be very critical to make knowledge discovery systems practically useful.

In this study, we collect several important types of bio-entity relationship information from manually annotated databases and literature, including protein–protein interactions, protein/gene regulations, protein–small molecule interactions, protein–GO term relationships, protein–pathway relationships, pathway–disease relationships and protein–species relationships. We further integrate the relationship information in a graph data structure, called integrated bio-entity network (IBN), where the vertices are bio-entities and edges are their relationships. Edges in IBN contain information on the types of the relationships, the directionalities of the relationships and the probabilities of the relationships. The rich information in the edges makes IBN a very effective system for knowledge discovery. To generate hypotheses automatically, we design graph-theoretic algorithms to extract high probability indirect relationships between bio-entities in the network. We show with examples that IBN can be used to generate plausible hypotheses for a given query, which can help researchers to better understand biological systems and design experiments.

## Results

### Data integration from databases and literature

#### Integration of bio-entity relationship information from databases

We first collect molecular interaction information from manually curated databases. For protein–protein interactions, BioGRID [5], EBI IntAct [11] and NCBI Gene database[42], [52] are used. Protein-small molecule interaction information is obtained from STITCH II database [13], [53], which is a collection of information from manually curated databases, high throughput experiments and text mining. Since we also extract protein–small molecule interaction information from literature using our own method[38], we filtered out those interactions with low scores in STITCH II database including those obtained from text mining.

In addition to molecular interaction information, we also collected other types of bio-entity relationships including protein–GO terms, protein–pathway, pathway–disease, and protein–species relationships. There have been some previous studies aiming to extract some of the above relationships automatically from literature[1], [24], [54], [55], [56], [57]. The relationship information between GO terms and proteins is obtained from Gene Ontology database[44] and GOA database[26], where such associations have been manually annotated for many of the GO terms. Relationships between pathways and proteins are obtained from pathway interaction database[58] and Reactome[59]. Relationships between diseases and pathways are obtained from KEGG database [60]. Totally, 30,707 GO terms, 607 pathway names, and 29,018 disease names are collected. 12,190 GO terms, 369 pathways, and 1,662 diseases are associated with at least one protein. 326,425 proteins are associated with at least one GO term. Among all 39,501 human proteins, 16,879 are associated with at least one GO term and 4,828 are associated with at least one pathway.

#### Large scale extraction of protein interaction information from literature

We performed large scale automatic extraction of protein-protein interaction information from literature including both physical interactions and regulatory information using a Bayesian network approach developed earlier[38]. All PubMed abstracts with at least one interaction word were downloaded and split into sentences to obtain triplets (two molecule names and one interaction word in a sentence constitute a triplet). Totally, we have 6,734,286 abstracts, 1,991,555 sentences with triplets, and 4,676,329 triplets. Among the extracted triplets, 652,236 are predicted as describing interactions, in which 335,176 are unique interactions. If only 40% of these predictions are true cases (estimated based on manually reading a small number of cases), there will be more than 130,000 new interactions added to the current 303,093 total interactions.

To extract protein–small molecule interaction (PSI) information, we obtained the small molecule name dictionary from NCBI PubChem database[61]. We filtered out protein–protein specific interaction words from the interaction word dictionary, such as *dimerization*, *phosphorylation* etc. Totally, we obtained 2,960,499 PSI triplets, and 505,060 are predicted as describing interactions using the BN model trained with protein-protein interactions[38]. We manually read ∼200 randomly selected predicted interaction triplets, which gave an estimated accuracy of about 35%. This accuracy is comparable to the best performance of current methods on general biological texts [62], [63] and showed that the method we developed for protein-protein interaction extraction can be readily extended to protein-small molecule interaction extraction.

### Bio-entity relationship integration and knowledge discovery

**Figure 1:**
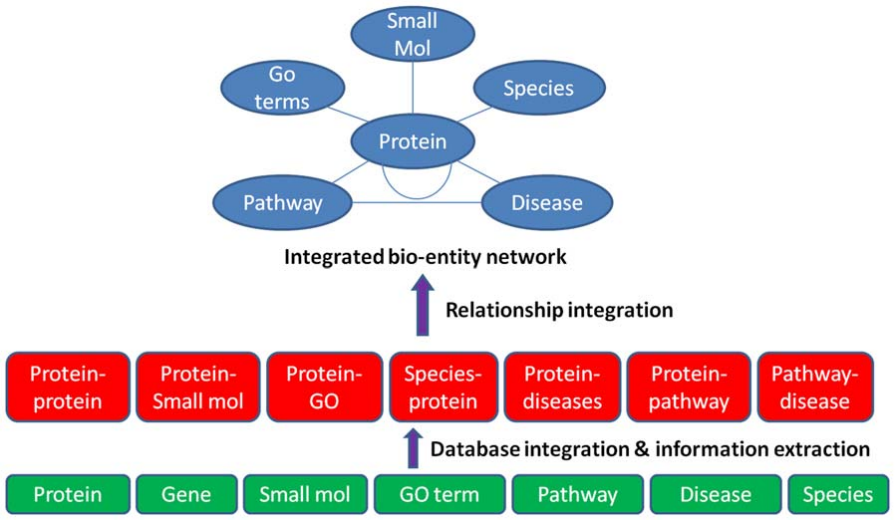
System overview. The knowledge discovery system for bio-entity relationships. Green boxes are bio-entities. Red ones are bio-entity relationships, which are used to build IBN.

With the relationships information collected, we would like to organize them in a structured form so that the power of integrated data can be harnessed conveniently and efficiently. Since all the relationships naturally form a network, graph representation is a compelling choice. We name the network formed by the relationships among bio-entities as integrated bio-entity network (IBN). Vertices or nodes in IBN represent bio-entities and edges represent relationships between bio-entities. Edges can be information confirmed manually such as those obtained from databases or extracted automatically from literature. Indirect links between two bio-entities through more than one edge may be valuable information that has not been documented in previous literature. Searching such information in IBN allows scientists to discover valuable new information. In fact, such practice has been done routinely by scientists in their research through a combination of manually reading the literature and performing searches on multiple databases. IBN thus can serve as a platform to assist researchers to automatically generate new hypotheses, which can be further tested through targeted experiments or literature review. The overview of the system is shown in Fig 1.

The generated hypotheses from IBN can be relationships between any two types of bio-entities in the network. For example, a researcher may want to find the effect of a small molecule to cells. Such effect can be a therapeutic effect of the small molecule to a disease or can be drug-side effect. To find such effects, the small molecule can be queried through IBN to retrieve all proteins, pathways, GO terms and diseases that related to this small molecule. Another typical question a researcher may ask is whether there is any relationship between two bio-entities he/she is interested in. In such case, the two bio-entities can be used as the input of a query that searches for all direct and indirect relationships with high probabilities between the two bio-entities. To perform the above searches, we designed graph theoretic algorithms, breadth-first search with pruning (BFSP) and most probable path (MPP) (see Methods for details). We illustrate how knowledge discovery can be performed using IBN through a few case studies.

#### Knowledge discovery case study 1: insulin network

**Figure 2:**
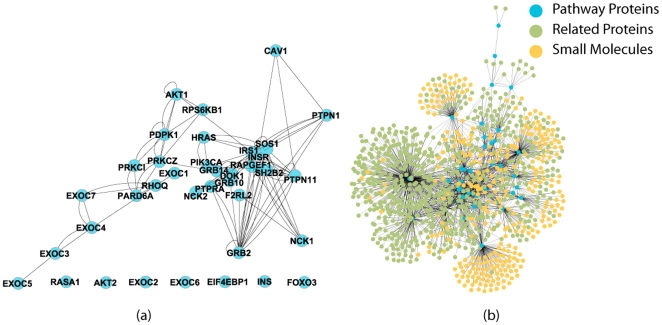
Insulin pathway network. a). Network with only proteins directly related to insulin pathway. b). Network with proteins, and small molecules. Nodes in blue, called pathway proteins, are proteins that are annotated as associated with insulin pathway as shown in a), nodes in green are proteins that interact with pathway proteins. Nodes in yellow are small molecules that interact with pathway proteins.

In this case study, we want to find all proteins related to insulin pathway directly or indirectly through other proteins. Using BFSP (see Method) starting with insulin pathway and retrieving only the bio-entities within two edges away from insulin pathway, the search returns more than two thousand interactions. The proteins directly related to insulin pathway are shown in Fig 2a and the proteins and small molecules that interact with them are shown in Fig 2b, where a subset of edges with probability *p* = 1 are selected. The molecular interaction information for insulin pathway, retrieved from IBN, not only shows how proteins within this pathway related to one another, but also shows how other proteins not directly associated with insulin pathway interact with those proteins. Some of those indirectly related proteins may actually be associated with insulin pathway, although they have not been annotated so far. For example, TRB3 was found to disrupt insulin signalling by binding to AKT[64]. In the current database, only AKT is annotated to be associated with insulin pathway. Based on the discovered information, we can add protein TRB3 to the list of proteins that are associated to insulin pathway. Another example is inhibitor kappaB kinase (IKK), which contributes to insulin resistance by phosphorylating protein IRS-1[65], a protein that has been annotated to be associated with insulin pathway. Again, protein IKK can be added to those proteins related to insulin pathway based on this information. The set of proteins that are currently annotated to be associated with insulin pathway is given in supplementary material (File S1). Retrieval of pathway related information can thus assist human annotation of protein-pathway associations. Other constraints can be easily incorporated into BFSP algorithm. For example, one can limit the proteins from human only, or limit the interaction relationships to be only one particular type, such as phosphorylation, inhibition or activation.

#### Knowledge discovery case study 2: aspirin network

**Figure 3:**
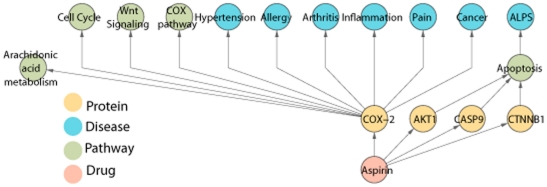
Aspirin network. Aspirin is connected to three proteins, which are connected to apoptosis pathway, which is then connected with disease ALPS. The network also provides a mechanism of action of aspirin as a treatment for ALPS.

We use aspirin as an example to illustrate how one can use IBN to search for diseases related to a small molecule. A BFSP search with aspirin and its synonyms as the query keyword and *p_c_* = 1 resulted in 144 proteins, where *p_c_* is the probability cutoff to prune away low probability relationships in BFSP algorithm. These are proteins that are known to directly interact with aspirin. Reducing *p_c_* to 0.5, we obtained 155 pathways and 1 disease (GO terms are ignored since they are not linked to diseases in IBN). Since the goal here is to find diseases related to aspirin, proteins indirectly interact with aspirin are ignored because they will not lead to a disease given *p_c_* = 0.5 (see BFSP procedure for probability calculations). The reason that only one disease was returned is because the only way we obtain disease information is through the aspirin-protein-pathway-disease route and pathway-disease relationship is poorly annotated in the current databases. The disease found is ALPS (Autoimmune Lymphoproliferative Syndrome). We did a literature search and did not find literature support for aspirin being a treatment for ALPS. In IBN, aspirin is connected to ALPS through pathway apoptosis and a few proteins associated with that pathway. The query result not only links aspirin to the disease ALPS, but also provides the edges that connect the two entities, which may shed light on the mechanism of the action of aspirin on the disease (if it is indeed effective). To find out more diseases related to aspirin, we did a text mining study using one of the proteins that is well known to interact with aspirin, Cox-2. We searched all PubMed abstracts for co-occurrences of pathway and disease names with Cox-2. We found that there are totally 444 diseases and 45 pathways. Some diseases co-occur with Cox-2 many more time than others, such as cancer (2585 co-occurrences) and pain (335 co-occurrences). Higher frequency of co-occurrence indicates higher likelihood of true association or stronger relationship. The diseases and pathways that are strongly associated with Cox-2, together with ALPS, are plotted in Fig 3.

#### Knowledge discovery case study 3: PMA network

PMA (phorbol ester, or 12-O-Tetradecanoylphorbol-13-acetate) is a potent tumor promoter often employed in biomedical research to activate the signal transduction enzyme, protein kinase C (PKC)[66]. PMA is also being studied as a drug in the treatment of hematologic cancer or bone marrow disorder and is currently undergoing phase 1 clinical trial[67]. In this study, our goal is to build a network around PMA that includes proteins, GO terms, and pathways that are affected by PMA directly or indirectly. Performing BFSP with PMA as the query keyword and *p_c_* = 0.5 returned thousands of proteins and interactions. This is not very surprising since many of proteins in PKC family and those regulating (or regulated by) them are hub proteins that are important in many biological processes. However, not all the reported proteins, pathways or GO terms are actually affected by PMA. The reason is that a significant part of the interaction information used by us is obtained from databases and there is no detailed interaction information available such as directions of the interactions. Proteins that are not affected by PMA directly or indirectly can also be returned, which is not desirable. Clearly, without the directionality information, many false positives are produced and the effect of the signal/query can be difficult to infer accurately.

**Figure 4:**
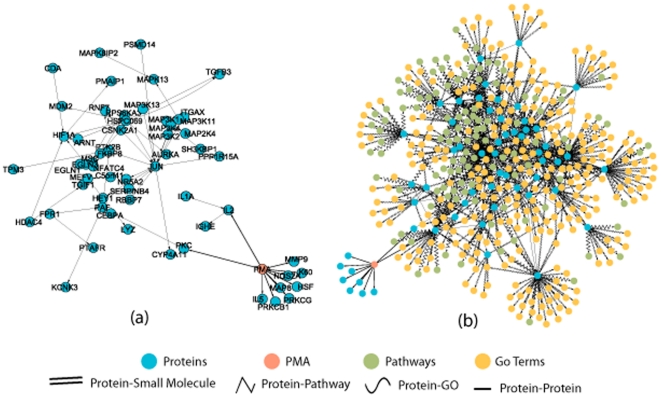
PMA network with direction information for the interactions. a) PMA and proteins. b) PMA, proteins, GO terms and pathways.

**Figure 5:**
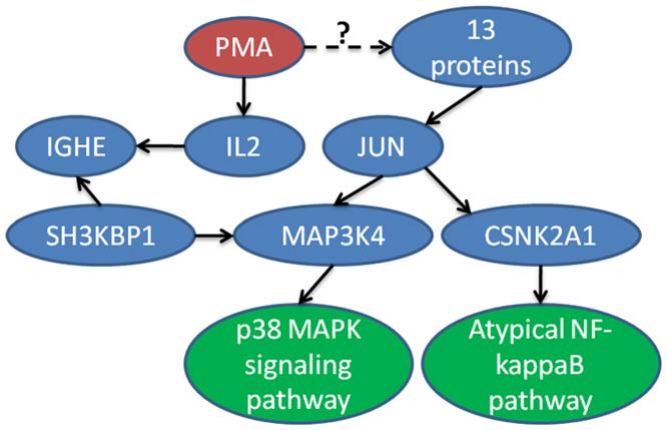
The most probable paths from PMA to two associated pathways.

We built a smaller network for PMA by requiring the interactions to be either regulatory type or phosphorylation using interactions extracted from literature, which resulted in only 79 proteins and 166 interactions in total. We manually verified the interactions and kept only the correct ones. The resulting directed network is shown in Fig 4a. In Fig 4b, pathways and GO terms associated with those proteins in Fig 4a are also shown. With this directed network, we can infer with more accuracy the pathways and GO terms affected by PMA. Some pathways are indeed found to be affected by PMA. For example, association of PMA with p38 MAPK signaling pathway is confirmed in Ref[68], and association of PMA with Atypical NF-kappaB pathway is confirmed in Ref[69]. The former was found through protein MAP3K4 and the latter was found through protein CSNK2A1. In both abstracts, there is no mentioning of the proteins, indicating the relationships were discovered indirectly through other literature. In Fig 5 we show the edges that link the pathways and PMA found using most probable path (MPP) algorithm (without taking directionality into account). Interestingly, the edges between PMA and the pathways do not actually explain the associations because the direction between IGHE and SH3KBP1 is the opposite of what one would expect. It is likely that the real mechanism is not through the path found by MPP. By looking at Fig 4, one can identify a few hub proteins and one of them, JUN, directly regulate the two proteins associated with the two pathways. JUN is also regulated by 13 other proteins. It is therefore tempting to speculate that the real pathway may go through JUN since regulation of any of the 13 proteins by PMA would give a plausible explanation of the associations between PMA and the two pathways. Further experiments can be designed using such information to elucidate the true mechanism.

**Figure 6:**
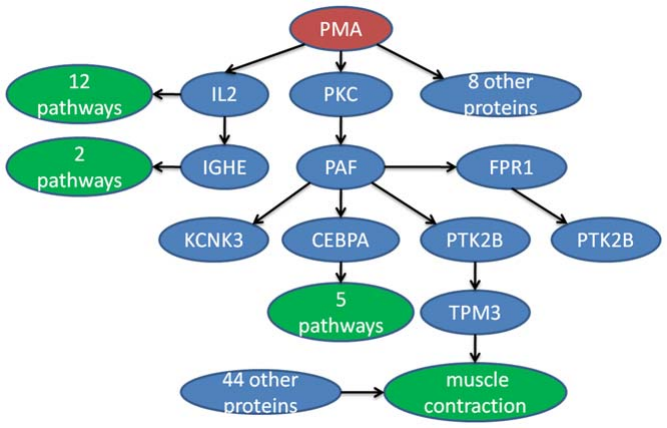
Proteins and pathways that are regulated by PMA directly and indirectly. PMA affected pathways through IL2 are: Calcineurin-regulated NFAT-dependent transcription in lymphocytes, IL27-mediated signaling events, IL12-mediated signaling events, IL23-mediated signaling events, Glucocorticoid receptor regulatory network, IL2-mediated signaling events, IL2 signaling events mediated by PI3K, Calcium signaling in the CD4+ TCR pathway, Regulation of Telomerase, IL12 signaling mediated by STAT4, Downstream signaling in naïve CD8+ T cells and IL2 signaling events mediated by STAT5; PMA affected pathways through IGHE are: IL4-mediated signaling events and Fc-epsilon receptor I signaling in mast cells; PMA affected pathways through CEBPA are: E2F transcription factor network, Regulation of retinoblastoma protein, regulation of Androgen receptor activity, FOXA2 and FOXA3 transcription factor networks and C-MYB transcription factor network; PMA affected pathway through TPM3 is Muscle contraction.

In Fig 6, we plot all the proteins and pathways that are affected by PMA directly or indirectly when directionality information is taken into account, which is substantially smaller than Fig 4b. Simply from the names of some of the pathways (given in the legend of Fig 6), we can see that they should be regulated by PMA since PMA directly activates IL2 and some of the pathways are related to IL2. Manual verification of all the relationships between PMA and pathways is not a trivial task. One way is to perform a retrieval search using both PMA and a pathway name as the keyword at PubMed. Returned articles from the searches can be manually read to confirm the relationship. For those PubMed searches with no hits, searches on Google sometimes provide clues on the relationship. Again those need to be carefully followed to confirm the relationship. For instance, searching PMA and pathway, Calcineurin-regulated NFAT-dependent transcription in lymphocytes, did not return any articles in PubMed. However, we found some evidence through Google search for the association at this URL: http://www.genome.jp/dbget-bin/www_bget?uniprot:O95644. Of course, even there is no any reported evidence for a relationship it can still be true.

Another pathway we examined is muscle contraction, which is separated from PMA by four proteins. PubMed search using PMA and muscle contraction as the keywords returned 182 articles. The first article[70] published recently studied the mechanism of PKC induced muscle contraction using mouse model and reported that PKC activation by PMA increased the level of protein TRPM4, which may be responsible for the smooth muscle cell depolarization and vasoconstriction of cerebral arteries. Using the PMA network in Fig 6 containing all the human proteins, another mechanism can be hypothesized, which can be tested experimentally if a follow-up by manual literature review considers it worthwhile. In the current database, muscle contraction is associated with other 44 proteins and PMA interacts with another 8 proteins. A manual database/literature search starting from those proteins to find the path between PMA and muscle contraction is clearly a daunting task.

## Discussion

In this study, we performed a large scale integration of a diverse set of bio-entities and their relationship information from both databases and literature and built a network based system, integrated bio-entity network (IBN), for biological knowledge discovery. We aim to address the three challenges faced by the current knowledge discovery studies, namely, data integration, relationship annotation and hypothesis ranking. Although there is still a lot of room for further improvement in all three areas, the framework we set up in this study presents a clear path toward effective automatic biological knowledge discovery. With the network data structure, graph theoretic algorithms can be designed to search for high probability indirect relationships (hypotheses) in IBN. Those automatically generated hypotheses based on the current knowledge base can help researchers to better understand their experimental results and design future experiments. A goal of future research would be to implement a publicly accessible knowledge discovery system.

Finally, we point out several directions that the current system can be further improved. Firstly, some relationship information is still poorly documented in the current databases such as protein-disease relationships and protein-pathway relationships. These relationships can be extracted automatically from literature[56], [71] and added to IBN. Secondly, relationship information needs to be specific to the particular relationship type and direction needs to be given where it is relevant. Such information can be obtained for interactions extracted automatically from literature. We recently developed a method similar to protein interaction extraction to predict the directionality of interactions and obtained very good accuracy (unpublished result). This method can be used to add directionality information to the edges in IBN. Thirdly, the probabilities associated with the relationships in IBN have been very helpful in estimating the probabilities of indirectly related bio-entities to rank the generated hypotheses. Estimation of the probabilities of automatically generated hypotheses can be further improved by building more sophisticated models using information of individual relationships. Finally, we want to point out that the protein naming system still needs to be improved. There are still a significant number of errors in annotated protein names.

## Methods

In this study, we use a previously developed protein interaction extraction method[38] to extract molecular interaction information from literature. The method is briefly described below. We first construct dictionaries containing words that are related to our information extraction task, including protein name dictionary, small molecule name dictionary, interaction word dictionary, GO term dictionary, pathway dictionary and disease dictionary. Abstracts that contain at least one interaction word are downloaded from PubMed database (up to Sept. 2009). The abstracts were then split into sentences. Sentences containing at least one triplet (two molecule names and one interaction word) are kept. Features are then extracted for each triplet in a sentence and parsed to a previously trained Bayesian Network (BN) model[38]. The model then estimates the probability of each triplet being a true interaction.

### Dictionaries

In information extraction, we use dictionaries to tag molecular names or interaction words. The synonyms of molecular names are incorporated in the dictionaries. All the synonyms of a molecule are linked to one vertex in IBN.

### Protein name dictionary

This dictionary contains totally 7,663,997 protein names. It was constructed by combining protein names from several sources including NCBI Gene database[42], [52], UniProtKB/Swiss-Prot[72] and BioThesaurus[73]. We filtered out false names in protein name dictionary using GENIES[74] and a large number of PubMed abstracts, where those names that are not tagged by GENIES as protein names in the PubMed abstracts more than half of the time (a rather conservative arbitrary cutoff) are filtered out. For example, “DNA replication” was a name in the original dictionary, but it was tagged as a protein name less than 20% of the time by GENIES among more than a thousand occurrences in the PubMed abstracts, so it was deleted from the dictionary.

#### Small molecule name dictionary

Obtained from NCBI PubChem database[61], this dictionary contains 38,791,284 names.

#### Interaction word dictionary

It contains words that describe interactions of molecules including regulatory relationships conftaining 192 words as used in the previous study[38].

#### GO terms

We obtained GO terms from Gene Ontology database[44]. There are totally 30,707 GO terms, which fall into three broad categories, molecule function, biological process, and cellular component. Some very common GO terms, such as “protein” are filtered out by a combined automatic and manual process.

#### Pathway names

We obtained pathway names from KEGG pathway database[60], Reactome[59], and pathway interaction database[58]. There are totally 607 pathways names. In the pathway database, some pathways have been annotated with relationship to certain disease and such information is used to infer relationship involving diseases.

#### Disease names

We obtained 29,018 disease names from PharmGKB[75]. Additional disease names were obtained from KEGG database.

#### Species names

We obtained species names from NCBI database. The protein–species relationships were obtained from UniProtKB/Swiss-Prot[72] and NCBI Gene database[42], [52].

### Graph theoretic algorithms and calculation of probabilities for indirect relationships

The probabilities of the relationships between any two vertices that are not connected by an edge in IBN can be calculated using the probabilities of existing edges. Any edge, representing a relationship between two bio-entities, has a probability assigned to it. For relationships obtained from manually annotated databases, the probabilities are 1. For relationships extracted from literature, the probabilities are given by the extraction method. When multiple instances are extracted for one particular relationship (i.e. several mentions of the same interaction between two proteins) from the literature, the highest probability among the instances is assigned as the probability for the relationship. Below we describe two algorithms that can be used to search for high probability relationships in IBN.

### Breadth-first search with pruning (BFSP) algorithm

To search for all indirectly connected vertices from a given vertex we perform a modified breadth-first search (BFS) algorithm[76], breadth-first search with pruning (BFSP), starting from the vertex. The BFSP procedure for a vertex *i* in a graph *G* is given below. Here we are only interested in vertices whose relationships to *i* have probabilities greater than a threshold value, *p_c_*. The additional pruning step aims to only include those significant relationships in the search result, which is essential in large scale knowledge discovery.

**procedure** BFSP(graph *G*, node *i*)

create a queue *Q*

enqueue vertex *i* onto *Q*

mark vertex *i*

**while***Q* is not empty

dequeue a vertex *v* from *Q*

**for each** unmarked neighbour *W* of *V*

**if***w* is not marked

*p_i_*_,*w*_  =  *p_i_*_,*v*_ × *p_v_*_,*w*_ × *p_d_*

/* *p_i,w_* is the probability for the relationship between node *i* and *w*, *p_i,v_* is the probability between node *i* and *v*, *p_v,w_* is the probability for node *v* and *w*, and *p_d_* is a parameter to model the uncertainty when inferring relationships through indirect edges */

**if***d_i,w_* > *p_c_*

/* *p_c_* is the threshold for selecting more relevant relationships */

mark *w*

enqueue *w* onto *Q*

In the above procedure the probability *p_d_* is used to model the uncertainty when inferring relationships through indirect edges. For example, even relationship between vertices A and B has probability 1 and that between vertices B and C also has probability 1, the relationship between A and C is not necessarily 1. In fact, in many cases such relationship can be false. For instance, even A interacts with B and B interacts with C, A may not interact with C. In principle, this probability can be learned from data and does not have to be a constant. In this study, we simply set *p_d_* to 0.8 and *p_c_* to 0.5, unless otherwise specified. This means all the indirect relationships involving more than three edges will not satisfy the *p_c_* threshold. In another word, the BFSP algorithm will not visit any vertices which are more than three edges apart from the query vertex.

#### Most probable path (MPP) algorithm

This algorithm is used to find the path between two bio-entities in IBN with the highest probabilities among all paths connecting the two bio-entities, which is based on Djikstra's shortest path algorithm[77].

**procedure** MPP (graph *G*, node *s*, node *t*)

/* initialize all the vertices in *G*(*V*, *E*), where *V* is the set of vertices and *E* is the set of edges */

for each vertex v in *V*

*p_v,s_*  =  infinity

pi[v]  =  nil

*p_s,s_*  =  1

/* *p_v,s_* is the probability of vertex *v* to *s*, pi[*v*] is the predecessor set of vertex *v* to *s*. */

*S*  =  {0} /* Make *S* empty */

*Q*  =  *V* /* Put the vertices in Q */

while not Empty(*Q*)

/* extract the vertex in Q which has the highest probability relationship with **s** */

*u*  =  ExtractMostProbable( *Q* );

if(*u*  =  =  *t*)

stop; /* the MPP between **s** and **t** has been found */

AddNode( *S*, *u* ); /* Add *u* to *S* */

/* checks whether the current best estimate of the MPP to *v* can be improved by

going through *u* */

for each vertex *v* in Adjacent( *u* )

if ( *p_v,s_* < *p_u,s_* × *p_u,v_* × *p_d_*) then

*p_v,s_*  =  *p_u,s_* × *p_u,v_* × *p_d_*

pi[*v*]  =  *u*

/* *p_u,v_* is the probability of the relationship between *u* and *v*, and *p_u,s_* is the probability of the relationship between *u* and *s*., *p_d_* is the probability modeling the uncertainty when inferring relationships from indirect edges as in BFSP. */

## Supporting Information

File S1 Supplementary material.
(DOCX)
