# Supplementary material for: Integrated Bio-Entity Network: A System for Biological Knowledge Discovery
Source: PLoS One. 2011 Jun 27;6(6):e21474. doi: 10.1371/journal.pone.0021474 (PMC3124513; doi:10.1371/journal.pone.0021474)
Supplement: File S1 — Supplementary material. (DOCX) [file pone.0021474.s001.docx]

Supplementary material

Integrated bio-entity network: a system for biological knowledge discovery

Lindsey Bell, Rajesh Chowdhary, Jun S. Liu, Xufeng Niu and Jinfeng Zhang

UniProtKB/Swiss-Prot IDs of proteins associated with insulin pathway.

P35568, P29353, P62993, P16333, Q9BX66, Q8TAG9, Q96A65, P42336, Q07889, Q06124, Q13322, P18433, P22681, P46108-1, Q15642, Q9UPT5, P20936, P31751, Q03135, Q9NPB6, Q05513, P17081, O00145, O43639, Q99704, O15530, Q14449, Q13905, O60645, Q9NV70, P01308, P31749, Q13541, P41743, Q96KP1, P06213, P27986, P01112, O00141, P18031, P23443, O14492, O00471, O00254, O43524

UniProtKB/Swiss-Prot IDs of proteins associated with muscle contraction pathway

P35609, Q92624, Q8IYI6, O15273, P19429, P13805, P11532, P35606, O43809, Q9Y5X1, P18848, Q9P209, P60660, O60504, Q9Y2I6, Q8NFF5, P38159, P18206, Q9NYB9, P19634, Q96KQ7, Q9UQB8, O60763, Q8NF64, Q8N684, P06753, O15372, Q9UHY1, Q9H1D0, Q14240, P33176, Q14164, P04179, Q96A65, P68366, P68133, P68032, Q9Y3C7, Q14896, Q9NWT8, Q14194, Q04759, P62736, P14649, P13535

UniProtKB/Swiss-Prot IDs of proteins regulated by PMA in the directed network

P45985, P01583, O75807, P05107, O15553, Q96B97, Q8N726, Q14289, Q9UBF6, Q92905, Q99759, P50479, Q16665, Q9NWT6, P06753, Q15583, Q9UBP5, P42772, Q13794, P05412, P32239, P56524, Q16576, Q86X73, O00487, P61626, O14965, P48594, Q8IUR6, Q13233, Q13301, P49715, Q9NYT8, Q9Y2U5, Q9P0L2, P61812, P10600, P17676, P05019, Q16584, Q15418, P51812, P25101, 50818, B5B2W9, Q13387, Q8WUQ3, P60568, O00482, P15391, Q7Z7C8, O43283, Q9Y6R4, O14649, O15264, P20702, Q9Y5J3, P32238, P25105, Q14318, P49716, P01854, Q9GZT9, Q15004, Q11206, Q6ZN53, Q9H6Z9, 28874, P68400, P32320, Q8IXM3, P21462, P27540, P57059, 387569, O60682, Q02928, P22362, Q13093
